# The Effect of Autophagy on Chronic Intermittent Hypobaric Hypoxia Ameliorating Liver Damage in Metabolic Syndrome Rats

**DOI:** 10.3389/fphys.2020.00013

**Published:** 2020-01-30

**Authors:** Fang Cui, Hao Fei Hu, Jing Guo, Jie Sun, Min Shi

**Affiliations:** ^1^Department of Electron Microscope Laboratory Centre, Hebei Medical University, Shijiazhuang, China; ^2^Department of Clinical Laboratory, Second Hospital of Hebei Medical University, Shijiazhuang, China

**Keywords:** chronic intermittent hypobaric hypoxia, metabolic syndrome, endoplasmic reticulum stress, autophagy, AMPK-mTOR signaling pathway

## Abstract

**Aim:**

Our previous study demonstrated that chronic intermittent hypobaric hypoxia (CIHH) can confer hepatic protection by reducing endoplasmic reticulum stress (ERS) in high-fat-high-fructose induced metabolic syndrome (MS) rats. It is known that there is a functional coupling between autophagy and ERS. This study aimed to investigate the effect of CIHH on autophagy function and adenosine mono-phosphate-activated protein kinase-mammalian target of rapamycin (AMPKα-mTOR) signaling pathway in hepatic tissue of MS rats.

**Main Methods:**

6-week old male Sprague-Dawley rats were randomly divided into: control (CON), CIHH (treated with hypobaric hypoxia simulating 5000-m altitude for 28 days, 6 h daily), MS (induced by 16-week high fat diet and 10% fructose water feeding), and MS + CIHH groups (exposed to CIHH after 16-week MS model). Food and water intakes, body weight, Lee’s index, fat coefficient, systolic arterial pressure, blood biochemicals, and histopathology of liver were measured, the expression of phosphorylated (p)-AMPK, p-mTOR, autophagy-related and ERS-related proteins were assayed in hepatic tissue.

**Key Findings:**

The MS rats displayed obesity, hypertension, polydipsia, glucose and lipids metabolism disorders, increased inflammatory cytokine, hepatic tissue morphological and functional damage, and the up-regulated expressions of ERS-related, autophagy-related proteins and p-mTOR, and the down-regulated expression of p-AMPKα. All aforementioned abnormalities in MS rats were ameliorated in MS + CIHH rats.

**Significance:**

In conclusion CIHH confers hepatic protection through activating AMPK-mTOR signaling pathway and the autophagy function, thus inhibiting ERS in hepatic tissue.

## Introduction

Metabolic syndrome (MS) is a metabolic disorder syndrome characterized by obesity, hyperlipidemia, atherosclerosis, hypertension, diabetes, etc. (Perez-Martinez et al., 2017). Recently, the proportion of obesity patients is rapid increasing worldwidely, and MS has become the first type of disease that endangers human health (Sinaga et al., 2018). As one of the key organ in body metabolism and energy storage, the liver is vulnerable to various stresses. Non-alcoholic fatty liver disease (NAFLD), the main expression of the MS and obesity in liver, promotes liver fibrosis in chronic liver diseases and increases the incidence of cirrhosis and liver cancer (Pagliassotti et al., 2016; Takaki et al., 2014; Zhang et al., 2014). Endoplasmic reticulum stress (ERS) mainly manifests as unfolded or misfolded protein accumulation. Studies have shown that ERS-mediated apoptosis is highly related to liver damage caused by viral hepatitis and NAFLD, which plays a crucial role in the pathogenesis of NAFLD (Lee et al., 2012; Zhang et al., 2012).

Autophagy is an evolutionarily conserved intracellular catabolic process that allows for the degradation and the turnover of damaged proteins and organelles in lysosomes. As one of essential housekeeping mechanisms to resist cell stresses (such as oxidative stress and ERS) and maintain intracellular homeostasis, autophagy is also known as autophagy flux for three major steps: autophagosome formation, fusion with lysosome, and eventual degradation, which is tightly regulated by many proteins encoded by autophagy-related genes (Yasuda-Yamahara et al., 2015). Moderate autophagy can inhibit ERS overactivation, reduce endoplasmic reticulum burden, and exert cytoprotective effects; while defective autophagy aggravates ERS, leads to defects in peripheral tissue insulin signaling pathways, which is associated with various diseases including cancer, neurodegenerative diseases as well as obesity-related cardio-metabolic diseases (De Meyer and Martinet, 2009).

The mammalian target of rapamycin (mTOR), which regulates cell growth and proliferation, maintains cellular energy homeostasis, and inhibits autophagy by inhibiting the formation of lysosomal and autophagy-related protein complexes. Adenosine monophosphate-activated protein kinase (AMPK), an upstream regulatory protein of mTOR, acts both as an upstream energy sensor and as a downstream autophagy activator. AMPK-mTOR signaling pathway is an important signaling regulatory pathway and plays an important role in autophagy.

Chronic intermittent hypobaric hypoxia (CIHH) simulates the plateau environment via low-pressure and low-oxygen conditions. Previous studies have shown that CIHH have beneficial effects on various tissues of the body (Cui et al., 2018; Shi et al., 2015; Tian et al., 2016; Yuan et al., 2015; Zhou et al., 2013), including improving ischemia/reperfusion or hypoxia/reoxygenation induced tissues damage (Zhou et al., 2013); improving endothelial dysfunction and vascular relaxation in mesenteric arteries (Cui et al., 2018); anti-inflammatory effect by reducing the level of collagen-induced arthritis inflammatory factors (Shi et al., 2015); improving fructose induced liver damage by inhibiting ERS (Yuan et al., 2015). However, the effect and mechanisms of autophagy on CIHH inhibiting ERS in the liver of rats, is still unclear. In this study, we tested the hypothesis that CIHH might ameliorate hepatic damage through improving AMPK-mTOR signaling pathway and autophagy, thus inhibiting ERS in high-fat-high-fructose induced MS rats.

## Materials and Methods

### Fructose Feeding and CIHH Treatment

6-week old male Sprague-Dawley rats (body weight: 80∼120 g) were provided by the Animal Center of Hebei Medical University. All experiments were carried out in compliance with the Guide for the Care and Use of Laboratory Animals as adopted and promulgated by the U.S. National Institutes of Health, and were reviewed and approved by the Ethics Committee for the Use of Experimental Animals in Hebei Medical University.

6-week old Sprague-Dawley rats (body weight: 80∼120 g) were randomly divided into four groups: control group (CON), CIHH treatment group, metabolic syndrome model group (MS), metabolic syndrome model plus CIHH treatment group (MS + CIHH). CON rats and CIHH rats were fed with chow diet (22% protein, 4% fat, and 50% carbohydrate; nutrient ratio, specific composition per 1,000 g: 99.50 g water, 216.97 g protein, 50.38 g fat, 56.87 g coarse ash, 24.00 g fiber, 13.29 g calcium, 9.17 g phosphorus) and drinking water. MS rats and MS + CIHH rats were fed with high fat diet (24% protein, 12% fat, and 42% carbohydrate; nutrient ratio, specific composition: 8% lard, 2% soy flour, and 90% chow diet) and water supplemented with 10% (wt/vol) fructose. Sixteen weeks later, CIHH and MS + CIHH rats were exposed to hypobaric hypoxia (simulation of 5000 m altitude for 28 days in a hypobaric chamber, 6 h/day, Po_2_ = 84 mm Hg), as described in our previous studies (Cui et al., 2018). All animals had free access to water and food and were housed in a temperature-controlled room (22 ± 1°C) with a 12 h/12 h light/dark cycle. During the experiment, body weight and systolic arterial pressure (SAP, Panlab model LE5001, Barcelona, Spain) were measured in conscious rats at a fixed time every week. During the 4 weeks of CIHH, the food and water intakes were also measured.

### Adipose Assay

At the end of the experiments, rats were fasted overnight and euthanized with a sodium pentobarbital overdose (50 mg/kg, intraperitoneal). Body weight and length were measured to calculate the Lee’s index [body weight × 1,000^1/3^/length (Miao et al., 2019). Mesenteric, epididymal, and perirenal fats were collected and weighted to derive the fat coefficient (fat weight/body weight) × 100%] (Shao et al., 2007).

### Blood Biochemical Assay

Blood samples were collected from the inferior vena cava of rats and centrifuged to get serum (3500 rpm, 10 min). Ultraviolet spectrophotometry was performed to determine the level of fasting blood glucose according to the principle of hexokinase reaction with automatic biochemical analyzer. Colorimetry was performed to determine the level of cholesterol, triglyceride, high density lipoprotein, low density lipoprotein, alanine transaminase (ALT), and aspartate aminotransferase (AST). Radioimmunoassay was performed to determine the level of insulin and homeostatic model assessment-insulin resistance (HOMA-IR) scores were calculated as fasting blood glucose × insulin/22.5 (Bonora et al., 2000). Enzyme-linked immunosorbent assay (ELISA) was performed to determine the level of interleukin6 (IL6) and tumor necrosis factor-α (TNF-α).

### HE Staining

Before collecting liver sample, the blood was removed through perfusing with saline (4°C) *in vivo*. A small segment of the hepatic tissue was fixed in 4% paraformaldehyde for 12 h, dehydrated and embedded in paraffin, cut into 4-μm-thick sections, stained with hematoxylin-eosin (HE), and then observed under an optical microscope (BX 50, Olympus Optical, Japan).

### Western Blot Analysis

The protein expression of AMPKα, phosphorylated (p)-AMPKα (Thr172), mTOR, p-mTOR (ser2448), 78KD glucose-regulated protein (GRP78), C/EBP-homologous protein (CHOP), Beclin-1, microtubule associated protein 1 light chain 3 (LC3), p62 was analyzed by Western blotting (Cui et al., 2018). Briefly, hepatic samples (100 mg) were collected and extracted for protein (centrifuged at 12000 rpm, 15 min, 4°C). All samples (20∼150 μg) were normalized according to the protein concentrations, separated by 7.5∼15% SDS-PAGE and transferred to polyvinylidene difluoride (PVDF) membranes. The blots were incubated with primary antibodies overnight at 4°C according to the instructions respectively. The reaction was visualized by the chemiluminescence, and the protein contents were normalized to GADPH.

### Chemicals

Fructose was purchased from AMRESCO (Solon, OH, United States). The kit for HE was purchased from Roche Applied Science (Indianapolis, IN, United States). BCA Protein Assay Kit was obtained from Tiangen Biotech (Beijing, China). Antibody against GRP78 (ab21685) was purchased from Abcam (Cambridge, United Kingdom). Antibody against p-AMPKα (Thr172, #2535), AMPKα (#5832), p-mTOR (Ser2448, #5536), mTOR (#2983), CHOP (#2895), Beclin-1 (#3495), and LC3 (#2775) were purchased from Cell Signaling Technology (Danvers, MA, United States). Antibody against p62 was purchased from Proteintech Group (Chicago, IL, United States). Antibody against GAPDH was purchased from Antibody Revolution (San Diego, CA, United States). All secondary antibodies were purchased from KPL (Gaithersburg, MD, United States). Enhanced chemiluminescence kit and PVDF membranes were obtained from Millipore Corporation (Billerica, MA, United States). ELISA kits were purchased from Shanghai Enzyme-linked Biotechnology Co., Ltd. (Shanghai, China).

### Statistical Analysis

All data were expressed as mean ± standard deviation (mean ± SD). One-way analysis of variance (one-way ANOVA) was used to compare multiple sets of data, and multiple comparisons between the two groups were performed using the Student-Newman-Keuls test (SNK test). *P* < 0.05 was considered to be statistically significant.

## Results

### Effects of CIHH on Body Weight, SAP, Food Intakes, Water Intakes, Lee’s Index and Fat Coefficient

**FIGURE 1:**
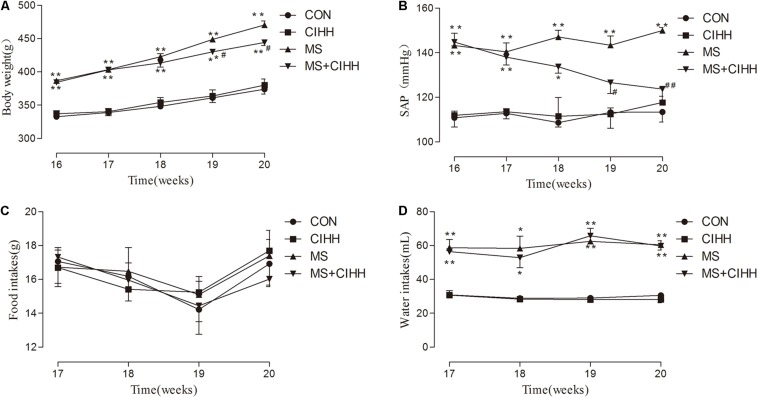
The effect of CIHH on body weight, systolic arterial pressure (SAP), food and water intakes. **(A)** The effect of CIHH on body weight. **(B)** The effect of CIHH on SAP. **(C)** The effect of CIHH on food intakes. **(D)** The effect of CIHH on water intakes. CON: control group, CIHH: CIHH group, MS: metabolic syndrome group, MS + CIHH: MS + CIHH group. All data were expressed as mean ± SD; *n* = 5–6 for each group. *P < 0.05 **P < 0.01 vs. CON, #*P* < 0.05 ##*P* < 0.01 vs. MS.

At the beginning of the experiment, body weight and SAP showed no statistical difference among four groups (*P* > 0.05). During the 16-week development of MS model, the body weights and SAP of the rats fed with the high-fat, high-fructose diet were heavier than those with the chow diet and drinking water (*P* < 0.05; Figures 1A,B), and in terms of the water intakes, the former were more than the latter (*P* < 0.01, Figure 1D), even though the food intakes were not different (*P* > 0.05, Figure 1C). During 4 weeks of CIHH treatment, there were not different in the food and water intakes of four groups rats, compared with before CIHH treatment. After 4 weeks of CIHH treatment, body weight, Lee’s index, fat coefficient and SAP were decreased in MS + CIHH rats compared with MS rats (*P* < 0.05, Figures 1A,B, and Table 1), while no significant difference between CIHH and CON rats (*P* > 0.05, Figures 1A,B, and Table 1). These data showed that CIHH could effectively antagonize polydipsia, obesity and hypertension in MS rats.

**TABLE 1:** Effect of CIHH on Lee’s index and fat coefficient.

	**CON**	**CIHH**	**MS**	**MS + CIHH**
Lee’s index	2.96 ± 0.10	2.92 ± 0.10	3.18 ± 0.04*	2.93 ± 0.04^#^
Fat coefficient	1.67 ± 0.18	1.64 ± 0.19	4.30 ± 1.04*	2.34 ± 0.85^#^

Lee’s index = body weight × 1,000 ^1/3^/length. Fat coefficient = fat weight (Mesenteric, epididymal, and perirenal fats)/body weight) × 100%. CON: control group, CIHH: CIHH group, MS: metabolic syndrome group, MS + CIHH: MS + CIHH group, All data are expressed as mean ± SD, *n* = 6 for each group, *P < 0.05 vs. CON, #*P* < 0.05 vs. MS.

### Effect of CIHH on Blood Biochemical Parameters

#### Effect of CIHH on Glucose and Lipid Metabolism

**FIGURE 2:**
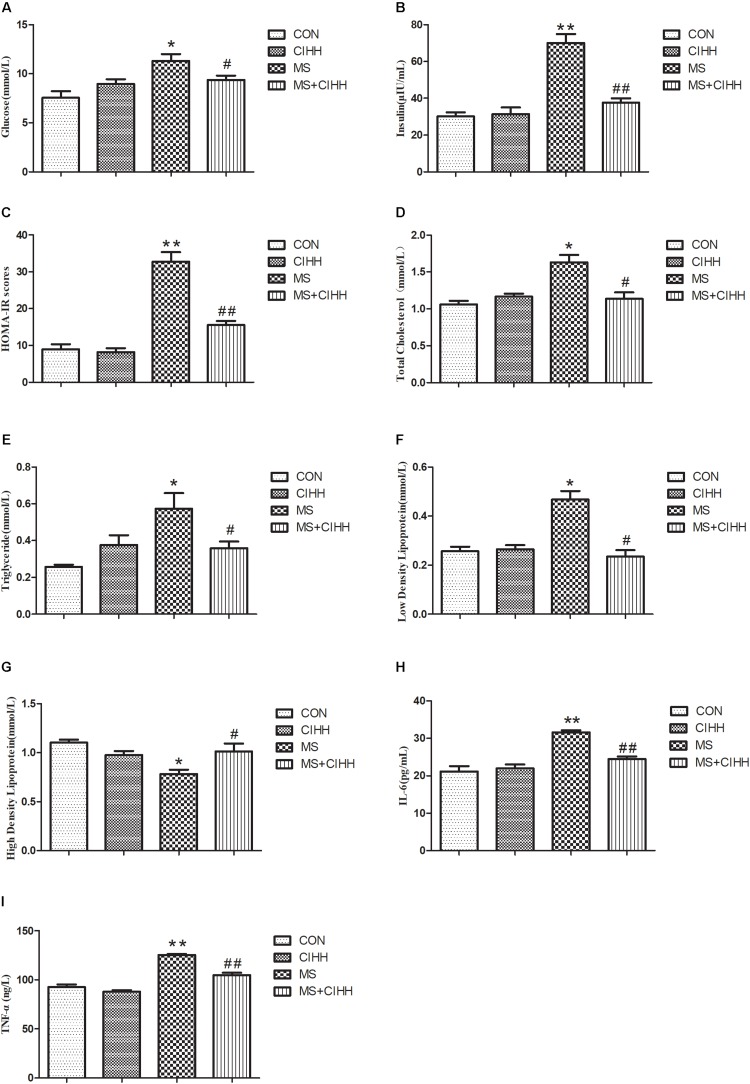
The effect of CIHH on blood biochemical parameters. **(A)** The effect of CIHH on glucose. **(B)** The effect of CIHH on insulin. **(C)** The effect of CIHH on homeostatic model assessment-insulin resistance (HOMA-IR) scores. **(D)** The effect of CIHH on total cholesterol. **(E)** The effect of CIHH on triglyceride. **(F)** The effect of CIHH on low density lipoprotein. **(G)** The effect of CIHH on high density lipoprotein. **(H)** The effect of CIHH on interleukin 6 (IL6). **(I)** The effect of CIHH on tumor necrosis factor-α (TNF-α). CON: control group, CIHH: CIHH group, MS: Metabolic syndrome group, MS + CIHH: MS + CIHH group. All data were expressed as mean ± SD; *n* = 6 for each group. *P < 0.05 **P < 0.01 vs. CON, #*P* < 0.05 ##*P* < 0.01 vs. MS.

Fasting blood glucose, insulin, HOMA-IR scores, cholesterol, triglyceride and low-density lipoprotein were significantly increased in MS rats compared with CON rats, and decreased in MS + CIHH rats compared with MS rats (*P* < 0.05, *P* < 0.01, Figures 2A–F). High-density lipoprotein was significantly decreased in MS rats compared with CON rats, and increased in MS + CIHH rats compared with MS rats (*P* < 0.05, Figure 2G). The results indicated that CIHH treatment could improve glucose and lipid metabolism disorders and insulin resistance in MS rats.

#### Effect of CIHH on the Level of Inflammatory Factor

Serum IL6 and TNF-α level was increased in MS rats compared with CON rats (*P* < 0.01) and decreased in MS + CIHH rats compared with MS rats (*P* < 0.01; Figures 2H,I). The results indicated that CIHH treatment could decrease inflammatory response in MS rats.

### Effect of CIHH on Hepatic Tissue

#### Effect of CIHH on Liver Morphology

**FIGURE 3:**
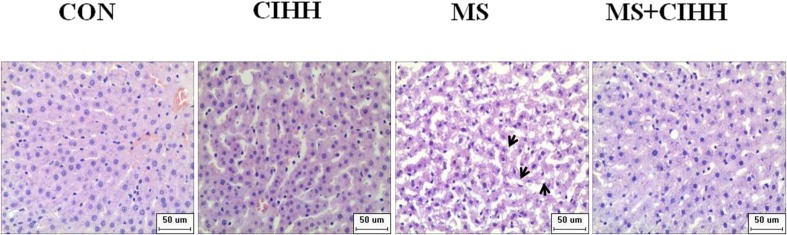
Effect of CIHH on pathologic morphology of liver. HE staining of liver (× 400), black arrows(↑) represent fatty degeneration and cord-like changes. *n* = 4 for each group.

As shown in Figure 3, HE staining revealed that the hepatic tissues of MS rats had obvious fatty degeneration and some cord-like changes compared with those of CON rats, while the morphology of hepatic tissues in MS + CIHH rats was significantly improved compared with MS rats. The results indicated that CIHH treatment could improve hepatic tissues morphological damage in MS rats.

#### Effect of CIHH on Liver Function

**FIGURE 4:**
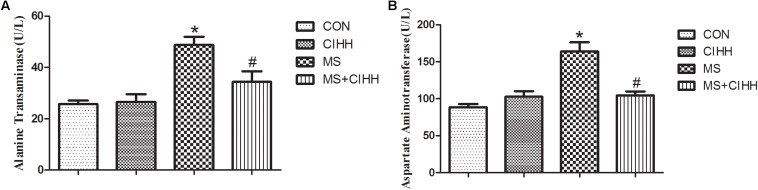
Effect of CIHH on the level of alanine transaminase **(A)** and aspartate aminotransferase **(B)**. CON: control group, CIHH: CIHH group, MS: metabolic syndrome group, MS + CIHH: MS + CIHH group. All data were expressed as mean ± SD; *n* = 6 for each group. *P < 0.05 vs. CON, #*P* < 0.05 vs. MS.

Compared with CON rats, the level of serum ALT and AST in MS rats was significantly increased (*P* < 0.05, Figure 4). Compared with MS rats, the level of serum ALT and AST in MS + CIHH rats was significantly lower (*P* < 0.05, Figure 4). The results indicated that CIHH treatment could improve hepatic tissues functional damage in MS rats.

### Effect of CIHH on Protein Expression

#### Effect of CIHH on Expression of ERS-Related Proteins

**FIGURE 5:**
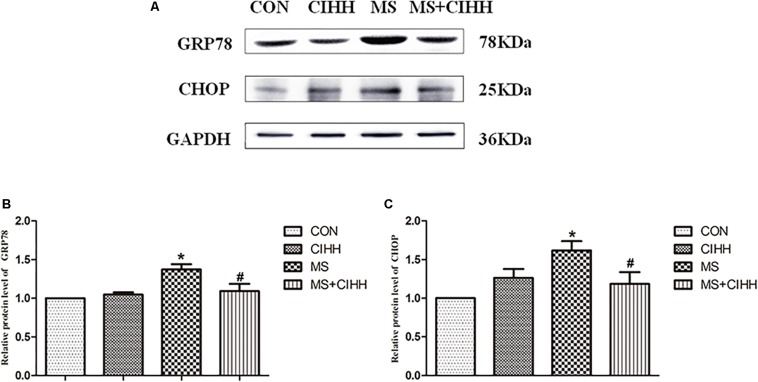
Effect of CIHH on the expression of ERS-related proteins in rat livers. **(A)** Original recording of Western blotting. **(B)** Quantification of protein levels of GRP78. **(C)** Quantification of protein levels of CHOP. CON: control group, CIHH: CIHH group, MS: metabolic syndrome group, MS + CIHH: MS + CIHH group. All data were expressed as mean ± SD; *n* = 4 for each group. *P < 0.05 vs. CON, #*P* < 0.05 vs. MS.

Compared with CON rats, the expression of ERS-related protein, GRP78 and CHOP, in liver tissues was significantly increased in MS rats (*P* < 0.05, Figure 5 and Supplementary Figure S1). The protein expression of GRP78 and CHOP in liver tissues was significantly decreased in MS + CIHH rats compared with MS rats (*P* < 0.05, Figure 5). The results indicated that the expression of ERS-related proteins was up-regulated in MS rats, and CIHH ameliorated the disturbance of ERS-related proteins in MS rats.

#### Effect of CIHH on the Expression of Autophagy-Related Proteins

**FIGURE 6:**
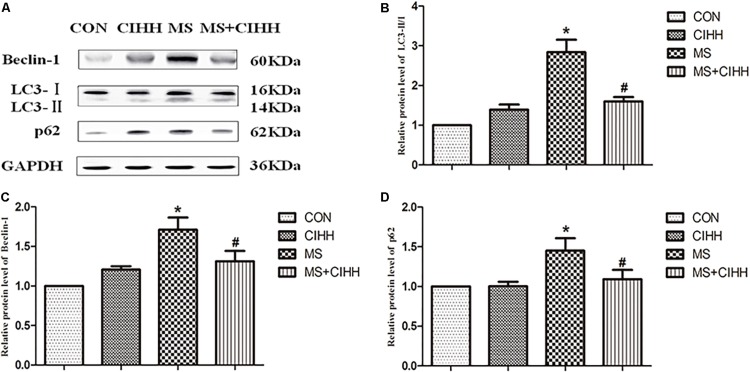
Effect of CIHH on the expression of autophagy-related proteins in rat livers. **(A)** Original recording of Western blotting. **(B)** Quantification of protein levels of LC3-II/I. **(C)** Quantification of protein levels of Beclin-1. **(D)** Quantification of protein levels of p62. CON: control group, CIHH: CIHH group, MS: metabolic syndrome group, MS + CIHH: MS + CIHH group. All data were expressed as mean ± SD; *n* = 4 for each group. *P < 0.05 vs. CON, #*P* < 0.05 vs. MS.

Compared with CON rats, the expression of Beclin-1, LC3-II/I and p62 in liver tissues was significantly increased in MS rats (*P* < 0.05, Figure 6 and Supplementary Figure S2). Compared with MS rats, the expression of Beclin-1, LC3-II/I and p62 in liver tissues was significantly decreased in MS + CIHH rats (*P* < 0.05, Figure 6). The results indicated that the expression of autophagy-related proteins was up-regulated in MS rats, but down-regulated in CIHH rats.

#### Effect of CIHH on the Expression of p-AMPKα and p-mTOR

Because autophagy is a dynamic process, the increased protein expression represents completely converse autophagy function: improvement or inhibition. Therefore, we further observed the effect of CIHH on one signaling pathway of inducing autophagy – AMPK-mTOR signaling pathway.

**FIGURE 7:**
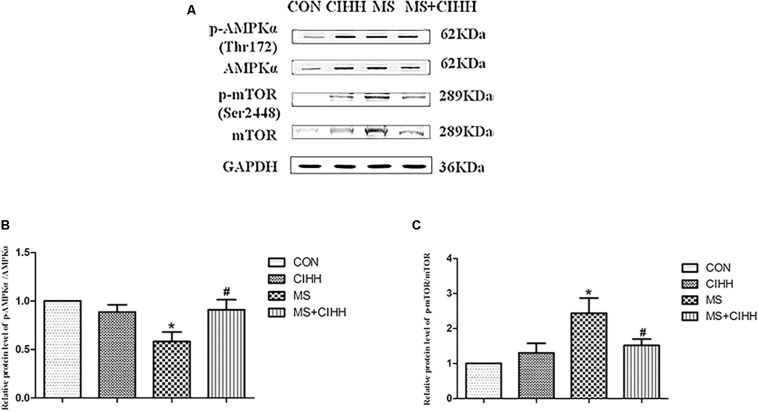
Effect of CIHH on the phosphorylation of AMPKα (Thr172) and mTOR (Ser2448) in rat livers. **(A)** Original recording of Western blotting. **(B)** Quantification of protein levels of p-AMPKα. **(C)** Quantification of protein levels of p-mTOR. CON: control group, CIHH: CIHH group, MS: metabolic syndrome group, MS + CIHH: MS + CIHH group. All data were expressed as mean ± SD; *n* = 4 for each group. *P < 0.05 vs. CON, *#P* < 0.05 vs. MS.

Compared with CON rats, the level of p-AMPKα (Thr172) was significantly down-regulated in MS rats (*P* < 0.05, Figure 7 and Supplementary Figure S3). Compared with MS rats, the level of p-AMPKα (Thr172) was significantly up-regulated in MS + CIHH rats (*P* < 0.05, Figure 7).

Compared with CON rats, the level of p-mTOR (Ser2448) was significantly up-regulated in MS rats (*P* < 0.05, Figure 7). Compared with MS rats, the level of p-mTOR (Ser2448) was significantly down-regulated in MS + CIHH rats (*P* < 0.05, Figure 7). The results indicated that the expression of p-AMPKα was down-regulated and the expression of p-mTOR was up-regulated in MS rats, and CIHH actived the AMPK-mTOR signaling pathway in MS rats.

## Discussion

The beneficial effect of CIHH on the impaired hepatocyte was investigated in high-fat-high-fructose induced MS rats. Compared with CON rats, MS rats showed polydipsia, obesity, hypertension, glucose and lipids metabolism disorders, increased inflammatory cytokine, liver tissue morphological and functional damage, and increased expression of ERS-related and autophagy-related proteins. The phosphorylation level of AMPKα was down-regulated while mTOR was up-regulated. CIHH treatment alleviated all changes in MS rats effectively, which suggested CIHH ameliorated hepatic damage through activating AMPK-mTOR signaling pathway, up-regulating autophagy, thus inhibiting ERS in MS rats.

MS is mainly characterized by obesity, hypertension, dyslipidemia, diabetes and insulin resistance. NAFLD is one of the main components and manifestation of MS which is used as the risk evaluation of type 2 diabetes and MS by the American Association of Clinical Endocrinologists (Pedro-Botet et al., 2018; Wang et al., 2006), because it further exacerbates other types of liver disease, such as viral hepatitis and alcoholic liver disease, as well as affects its therapeutic effect (Corte et al., 2015). CIHH simulates the plateau environment of low-pressure and hypoxic conditions, and the experimental animals are placed in this environment discontinuously. Our previous studies have shown that CIHH can improve rat liver injury induced by fructose feeding by inhibiting ERS (Yuan et al., 2015). In this study, MS rats fed with 16 weeks high-fat-high-fructose diet had pathological manifestations, liver damage, and can be significantly improved after 28 days of CIHH treatment.

The endoplasmic reticulum is an organelle in the cell which main function is to synthesize, modify, and fold proteins, store calcium ions, and regulate intracellular calcium ion balance. ERS is induced to degradate the accumulation of the wrong protein in the lumen of the endoplasmic reticulum for the maintenance of cell normal function. It is the main causes of NAFLD, that insulin resistance, oxidative stress, obesity and dyslipidemia, cause inflammation and infiltration of simple fatty liver and hepatocytes, and eventually lead to liver fibrosis and cirrhosis (Sangouni and Ghavamzadeh, 2019). Nakatani (Nakatani et al., 2005) found that the development of insulin resistance was closely related to ERS which promote the aggravation of insulin resistance and the insulin resistance of extrahepatic tissues. At the same time, hepatocyte injury is closely related to ERS-mediated apoptosis. In patients with alcoholic fatty liver and steatohepatitis, there is obvious hepatocyte apoptosis, and the degree of apoptosis is related to the severity of the disease and liver fibrosis. Thus, studies have shown that patients with diabetes, high-fat diet, and insulin resistance are more likely to develop ERS than normal individuals. ERS may be an important link in the process of liver injury (Nakatani et al., 2006).

GRP78 is a molecular chaperone of the endoplasmic reticulum (Su et al., 2011; Zhao et al., 2014), dissociated from the three stressor proteins on the endoplasmic reticulum membrane (Nakatani et al., 2005) to promote proper folding, assembly and transport of proteins, modification during the activation of ERS. When ERS lasts for a longer time or is more severe, it not only will be compensated by up-regulated expression of GRP78, but also induces the apoptotic pathway and apoptosis-related molecules, such as CHOP, caspase-12, JNK, etc. Therefore, GRP78 and CHOP are the hallmark proteins of ERS (Su et al., 2011; Zhao et al., 2014). Compared with normal-fed KKAy mice and control C57BL/6J mice, the mRNA levels of ERS marker genes GRP78 and CHOP were significantly elevated in white adipose tissue of high-fat diet mice. In the study we found that the up-regulation of GRP78 and CHOP was attenuated by CIHH. It indicated that CIHH may protect against hepatic damage by ameliorating ERS in hepatic tissue of MS rats.

ERS and autophagy interact and are closely related. ERS induces autophagy to degradate the excessive accumulation of unfolded or misfolded proteins and restore the endoplasmic reticulum homeostasis (Chandrahas et al., 2018; Ogata et al., 2006), which is called as endoplasmic reticulum actived autophagy (ERAA); on the other hand, autophagy can also affect ERS. For example, inhibition of autophagy with chloroquine can aggravate ERS and increase cell death. Autophagy participates in the degradation of lipids and lipid metabolism in hepatocyte, maintains fat homeostasis, and is closely related to the development of fatty liver and insulin resistance. Studies have shown that the up-regulation of autophagy promoted liver fat removal, while the down-regulation lead to the accumulation of lipids. In high-fat diet, the accumulation of lipids in hepatocytes will down-regulate autophagy and further aggravate the accumulation of lipids in hepatocyte, thus form a vicious circle of liver damage and the development of steatohepatitis (Seth et al., 2011). Autophagy can provide survival protection for liver cancer cells in the ERS state, and has no significant effect on normal liver cells. In the study we found that the expression of autophagy-related proteins LC3-II/I, Beclin-1 and p62 was increased in MS rats, and was decreased by CIHH treatment. Because autophagy is a dynamic process, the increased protein expression represents two different states of autophagy: increased autophagosome synthesis indicates an increase in autophagy function; and blocked degradation indicates a decrease in the autophagy function. Therefore, we further observed the effect of CIHH on one signaling pathway of regulating autophagy – AMPK-mTOR signaling pathway.

The AMPK-mTOR signaling pathway plays an important role in the regulation of autophagy. AMPK, an energy receptor, is a key regulatory site in endocrine diseases such as obesity and diabetes, and activates autophagy by inhibiting mTOR activity. Under normal conditions, AMPK remains dephosphorylated and inactive state; when energy is shortage, such as obesity and MS, AMPK is phosphorylated at the site of Thr172 and actived, which directly phosphorylates the Thr2446 site of mTOR, inhibits Akt-mediated phosphorylation of Ser2448, indirectly inhibits mTOR activity, thus activates autophagy. Studies have shown that high-fat, high-fructose-induced obese mice and rats have abnormal AMPK-mTOR signaling pathways, and vascular endothelial AMPK phosphorylation levels are significantly reduced, while mTOR phosphorylation levels are significantly increased (Ma et al., 2010). In the study we found that CIHH could increase the down-regulated phosphorylation level of AMPKα, and decrease the up-regulated phosphorylation level of mTOR, which suggested that CIHH could activate AMPK-mTOR signaling pathway, then up-regulate the autophagy function in hepatocyte tissue of MS rats. In the light of increased autophagy-related protein expression in MS rats, we speculated that the accumulation of autophagy-related proteins may be caused by blocked autophagy degradation and defective autophagy function, and it warrants further investigation in future studies.

CIHH as a training or treatment method for sports and cardiovascular diseases has been reported in former Soviet Union decades ago, and has been proved having lots of beneficial action such as losing of body mass and improvement of metabolic abnormality (Kayser and Verges, 2013). With optimal level and time, CIHH is promising to become a potential therapy for prevention and treatment of metabolic and cardiovascular dysfunctions in obese and MS patients.

## Conclusion

In conclusion, our study demonstrates for the first time that CIHH confers hepatic protection in high-fat-high-fructose induced MS rats, which might be related to the activation of AMPK-mTOR signaling pathway and the autophagy function, thus the inhibition of ERS.

## Data Availability Statement

All datasets generated for this study are included in the article/Supplementary Material.

## Ethics Statement

The animal study was reviewed and approved by the Ethics Committee for the Use of Experimental Animals in Hebei Medical University.

## Author Contributions

FC, HH, and JG performed the experiments and drafted the manuscript. JS analyzed the data. MS was responsible for conception and design of the research, revised, and approved the final manuscript.

## Conflict of Interest

The authors declare that the research was conducted in the absence of any commercial or financial relationships that could be construed as a potential conflict of interest.
