# Supplementary material for: The Effect of Autophagy on Chronic Intermittent Hypobaric Hypoxia Ameliorating Liver Damage in Metabolic Syndrome Rats
Source: Front Physiol. 2020 Jan 30;11:13. doi: 10.3389/fphys.2020.00013 (PMC7002389; doi:10.3389/fphys.2020.00013)
Supplement: Supplementary file 1 [file Data_Sheet_1.PDF]

Supplementary information

The effect of autophagy on chronic intermittent hypobaric hypoxia ameliorating liver damage in metabolic syndrome rats

Fang Cui[2*], Hao-Fei Hu[1*], Jing Guo[1], Jie Sun[1], Min Shi[1**]

1 Department of Clinical Laboratory, The Second Hospital of Hebei Medical University, Shijiazhuang 050000, PR China

2 Department of Electron Microscope Laboratory Centre, Hebei Medical University, Shijiazhuang 050017, PR China

* These authors contribute this study equally

**corresponding author：Min Shi, MD, PhD

Department of Clinical Laboratory, The Second Hospital of Hebei Medical University, Shijiazhuang, Hebei Province, 050000, China. Tel: +86-311-66003770 (Office). E-mail: sm8344@sina.com

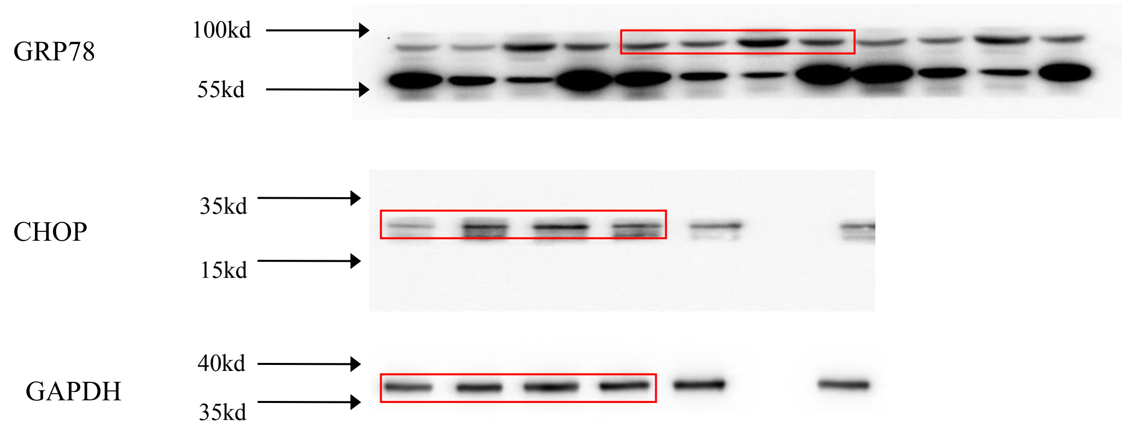

Supplementary Figure S1

Primary data of full-length blots of Figure 5. The samples (CHOP: 20 μg; GRP78: 50 μg) were examined by 15% SDS-PAGE, and transferred to PVDF membranes which were cut into several parts according to molecular weight. The exposure time varies with instruction and the image effect, specifically: GRP78 20 sec; GAPDH 1 min; and CHOP 3 min. Boxes indicate lanes which were used in Figure 5 of manuscript. The grouping of blots were cropped from different gels, and processed by changing brightness and contrast.

Fig.6

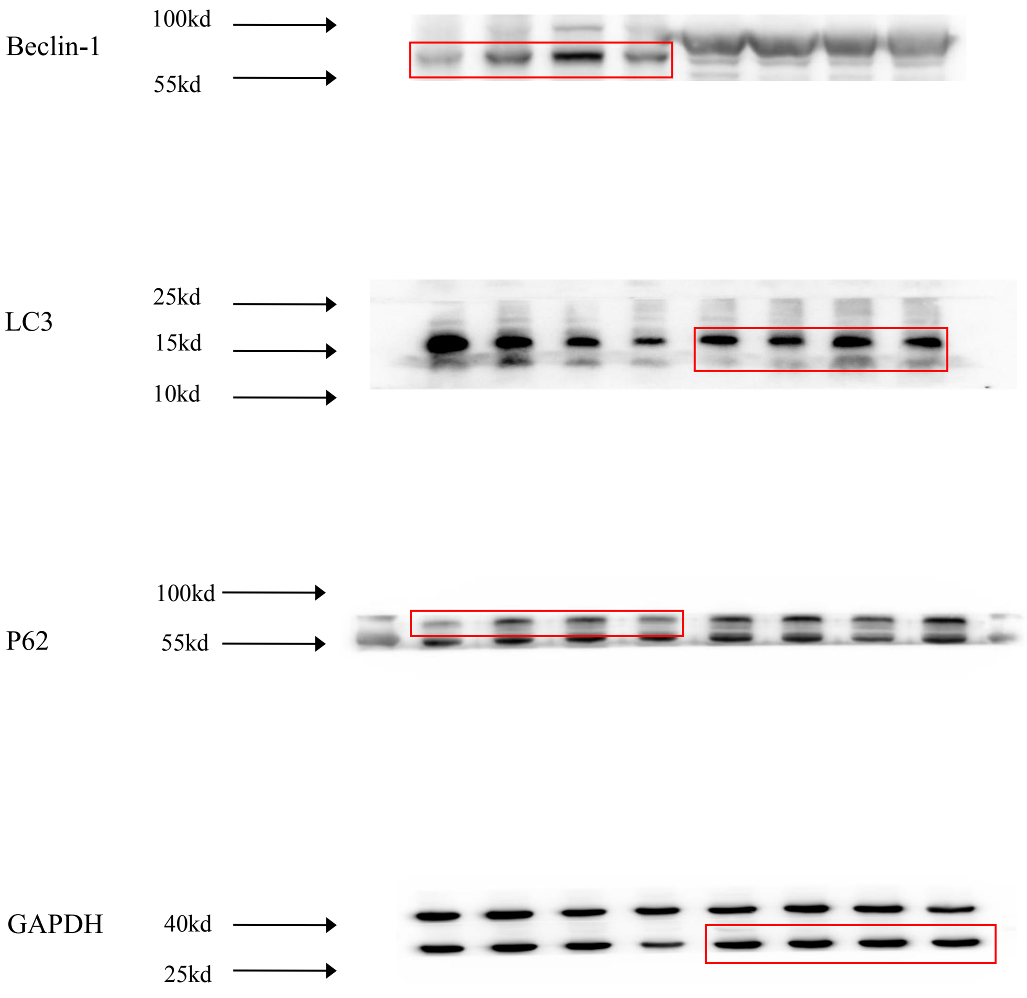

Supplementary Figure S2

Primary data of full-length blots of Figure 6. The samples (LC3B: 20 μg; Beclin-1: 50 μg) were examined by 15 % SDS-PAGE, and transferred to PVDF membranes which were cut into several parts according to molecular weight. The exposure time varies with instruction and the image effect, specifically: GAPDH 1 min; Beclin-1 1.5 min; LC3B: 2 min. Boxes indicate lanes which were used in Figure 6 of manuscript. The grouping of blots were cropped from different gels, and processed by changing brightness and contrast.

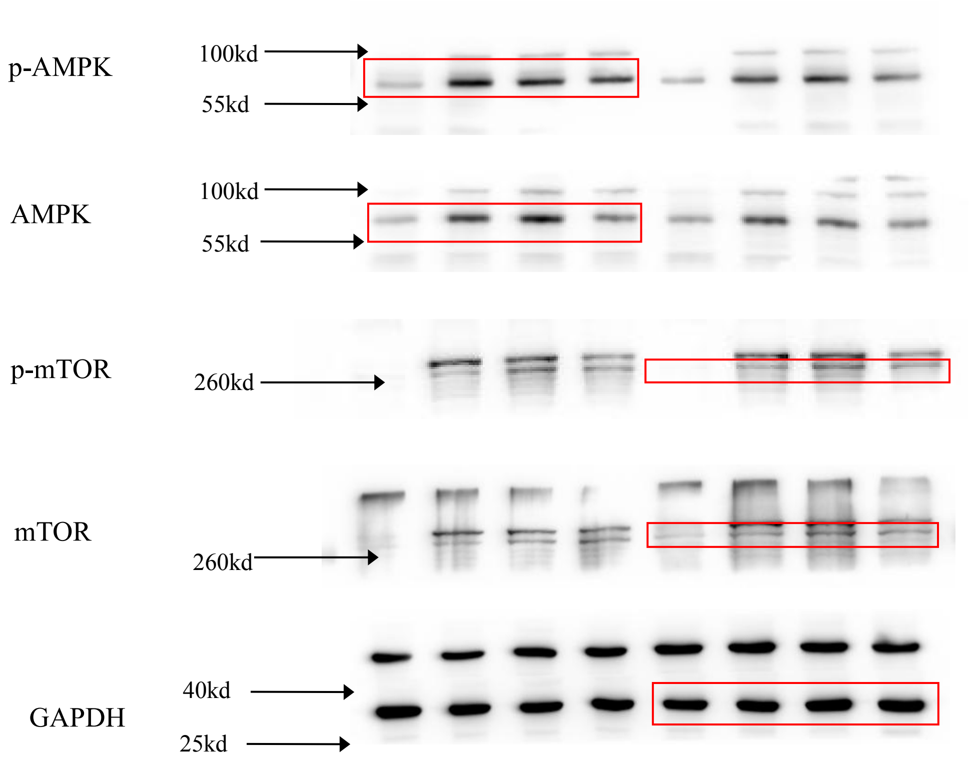

Supplementary Figure S3

Primary data of full-length blots of Figure 7. The samples (p-mTOR and mTOR: 150 µg; p-AMPK and AMPK: 50 µg) were examined by 7.5 % (p-mTOR and mTOR) and 15 % (p-AMPK, AMPK and GAPDH) SDS-PAGE respcetively, and transferred to PVDF membranes which were cut into several parts according to molecular weight. The exposure time varies with instruction and the image effect, specifically: GAPDH 1 min; p-AMPK and AMPK: 2 min; p-mTOR and mTOR: 2.5-3 min. Boxes indicate lanes which were used in Figure 7 of manuscript. The grouping of blots were cropped from different gels, and processed by changing brightness and contrast.
